# Asbestos exposure and haematological malignancies: a Danish cohort study

**DOI:** 10.1007/s10654-020-00609-4

**Published:** 2020-02-10

**Authors:** Else Toft Würtz, Johnni Hansen, Oluf Dimitri Røe, Øyvind Omland

**Affiliations:** 1 Department of Occupational and Environmental Medicine, Danish Ramazzini Centre, Aalborg University Hospital, Havrevangen 1, 4th, 9000 Ålborg, Denmark; 2 Danish Cancer Society Research Center, Strandboulevarden 49, 2100 Copenhagen Ø, Denmark; 3 Department of Clinical Research and Molecular Medicine, Norwegian University of Science and Technology, Prinsesse Kristinasgt. 1, Gastrosenteret 3rd, 7491 Trondheim, Norway; 4 Department of Clinical Medicine, Clinical Cancer Research Center, Aalborg University Hospital, Sdr. Skovvej 15, 9000 Ålborg, Denmark; 5 Department of Clinical Medicine, Aalborg University, Sdr. Skovvej 15, 9000 Ålborg, Denmark

**Keywords:** Childhood exposure, Environmental exposure, Job exposure matrix, Leukaemia, Lymphoma, Occupational exposure

## Abstract

Environmental asbestos exposure and occupational asbestos exposure increase the risk of several types of cancer, but the role of such exposures for haematological malignancies remains controversial. We aimed to examine the risk of haematological malignancies: first, in subjects exposed early in life, independently of any occupational exposure occurring later; second, in subjects exposed occupationally. We established an environmentally exposed cohort from four schools located near the only former asbestos cement production plant in Denmark. We identified nearly all pupils in the seventh grade and created an age and sex-matched 1:9 reference cohort from the Danish Central Population Register. Participants were born 1940–1970 and followed up in national registers until the end of 2015. Occupational asbestos exposure was assessed for all participants using two different job exposure matrices. The school cohort included 12,111 participants (49.7% girls) and the reference cohort 108,987 participants. Eight subgroups of haematological malignancy were identified in the Danish Cancer Registry. These cases were analysed for combined overall haematological malignancy, a combined subgroup of lymphomas and a combined subgroup of leukaemias. The data were analysed using Cox regression (hazard ratios (HR)) including other cancers and death as competing risks. Haematological malignancy was identified in 1125 participants. The median follow-up was 49.3 years (0.1–63.4). Early environmental asbestos exposure was not associated with an increased risk of haematological malignancy. Long-term occupational asbestos exposure was associated with overall haematological malignancy (HR 1.69, 95% CI 1.04–2.73); in particular for the leukaemia subgroup (HR 2.14, 95% CI 1.19–3.84). This large follow-up study suggests that long-term occupational asbestos exposure is associated with increased leukaemia risk. However, further studies are needed to confirm these observations.

**Electronic supplementary material:**

The online version of this article (10.1007/s10654-020-00609-4) contains supplementary material, which is available to authorized users.

## Introduction

Haematological malignancies form a heterogeneous group of diseases with diverse incidence, prognosis and aetiology [1]. Asbestos fibres have previously been found in bone marrow of acute myelocytic leukaemia patients [2]. Asbestos exposure is a confirmed carcinogen [3]. It has no established role for haematological malignancies, although an association has been suggested in case reports with different haematological malignancy outcomes in past decades [2, 4, 5]. Subsequent epidemiological studies and reviews have reported inconsistent results [6–8]. Asbestos is the common name of six commercially regulated minerals out of the approximately 400 naturally occurring fibrous silicate minerals [9, 10]. Asbestos is the key risk exposure for malignant mesothelioma but is also related to other primarily respiratory cancer types, e.g. lung and larynx cancer [11]. To our knowledge, no large epidemiological study on asbestos exposure and haematological malignancies has been conducted. In Denmark, such studies are feasible due to the well-established long-term high-volume use of asbestos in a Danish local asbestos cement plant combined with the existence of complete and high-quality national registers [12–14].

Asbestos was used in Denmark for at least six decades until the adoption of a national ban on asbestos in 1986 [15]. The Danish Eternit Fabric Ltd (DEF) was the only asbestos cement production plant in Denmark operating from 1928. Considerable information about the asbestos use in the DEF was presented in a paper by Raffn et al. (1989), describing the incidence of cancer and mortality among the employees [16]. The DEF used approximately 90% (~ 620,000 tons) of all imported asbestos for production of asbestos cement building materials in Denmark. The used asbestos type was mainly chrysotile from Cyprus (89%), supplemented with amosite (10%) and crocidolite (1%) from 1946 and 1952, respectively [16]. The DEF along with a lime pit source used in the asbestos cement production were located in the city of Aalborg. Air photos from the 1950s show an urban high-density housing next to the DEF. As asbestos cement production was primarily mechanical with a low technical standard [16], substantial amounts of waste asbestos fibres from the production site were released to the environment. The transport of asbestos from the harbour to the DEF in jute bags and the low-tech nature of the asbestos cement production caused the release of a substantial amount of asbestos to the environment as well; our best guess is 1–10%, which is equal to 6200−62,000 tons of asbestos over the years. Building products produced at the DEF had an asbestos fibre content of 5–20% [16]. Estimates of the amount of airborne asbestos fibres inside the DEF varied from 50–800 fibres f/ml in 1948 to 10–100 f/ml in 1957 [16]. More than 41% of the person samples measured inside the plant in 1973 exceeded the time threshold limit value of 2 f/ml [16, 17]. The Danish threshold limit value for asbestos is now 0.1 fibre/cm^3^ [18]. To our knowledge, the extent of fibre exposure outside the plant was never assessed. Importantly, an increased risk of mesothelioma was found in the same school cohort, validating that there was environmental waste of asbestos which could have initiated the development of cancer [19].

We hypothesised that children living and attending schools in the vicinity of the asbestos cement plant had an environmental asbestos exposure early in life that could increase their risk for haematological malignancies later in life. Two different job exposure matrices (JEM) were used to provide a rough classification of occupational asbestos exposure in order to distinguish the effect of childhood environmental exposure from that of occupational exposures occurring in adulthood.

## Methods

### Environmental exposure

**Fig. 1:**
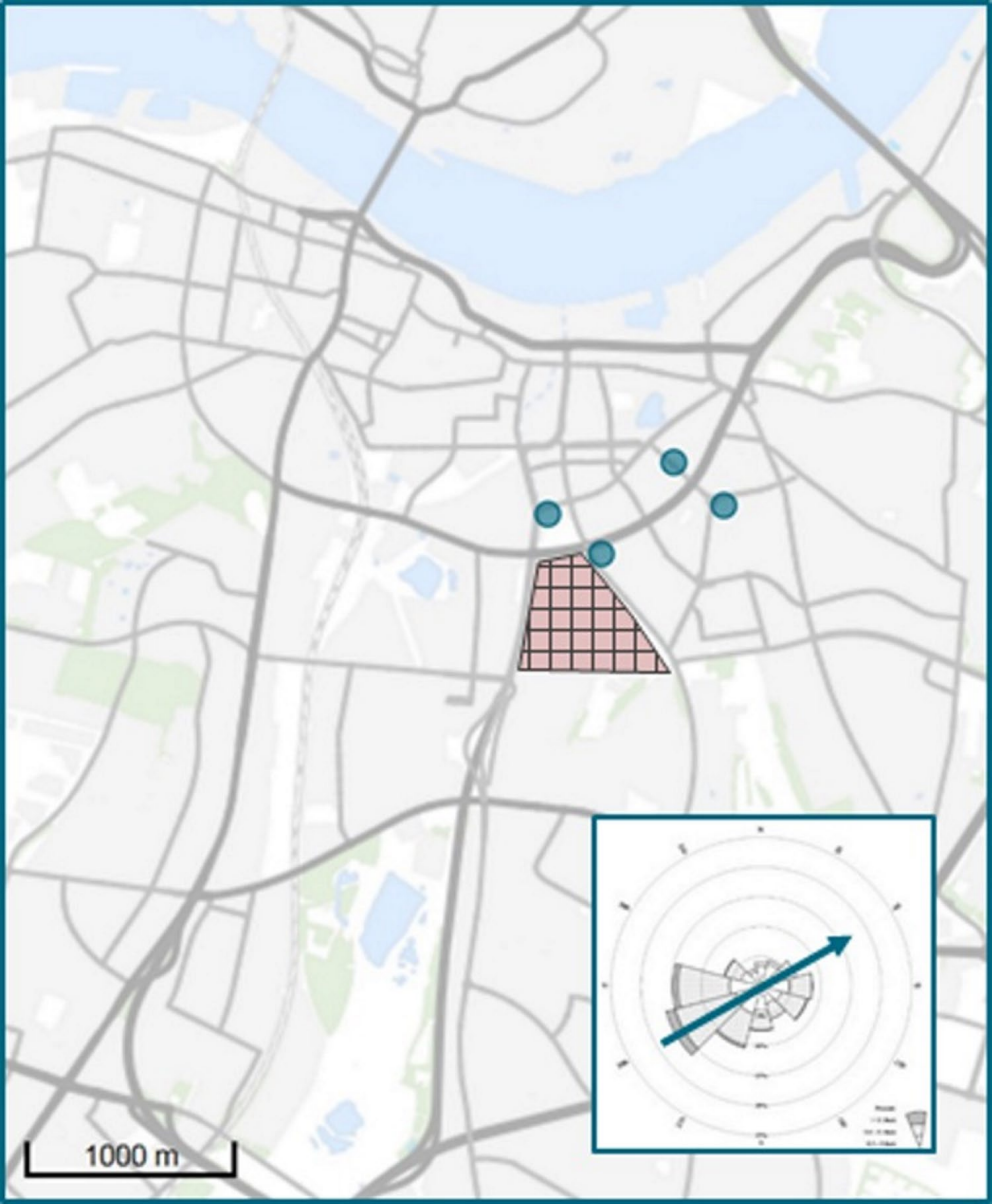
A section of the Aalborg city map, Denmark. * Note* Check pattern, the asbestos cement plant ‘Danish Eternit Fabric Ltd’; Dots, the four schools; Square right bottom, Aalborg wind rose with an arrow indicating the dominant wind direction west-southwest

To assess the impact of environmental asbestos exposure, we used old school records to identify children attending schools and living in the area close to the DEF. We selected schools located in the dominant wind direction in Aalborg, i.e. west-southwest of the DEF [20], assuming that these schools were the ones to be most contaminated by environmental asbestos fibres. Four primary schools were identified, located at a distance of 100–750 m from the DEF (Fig. 1).

### Study population

**Fig. 2:**
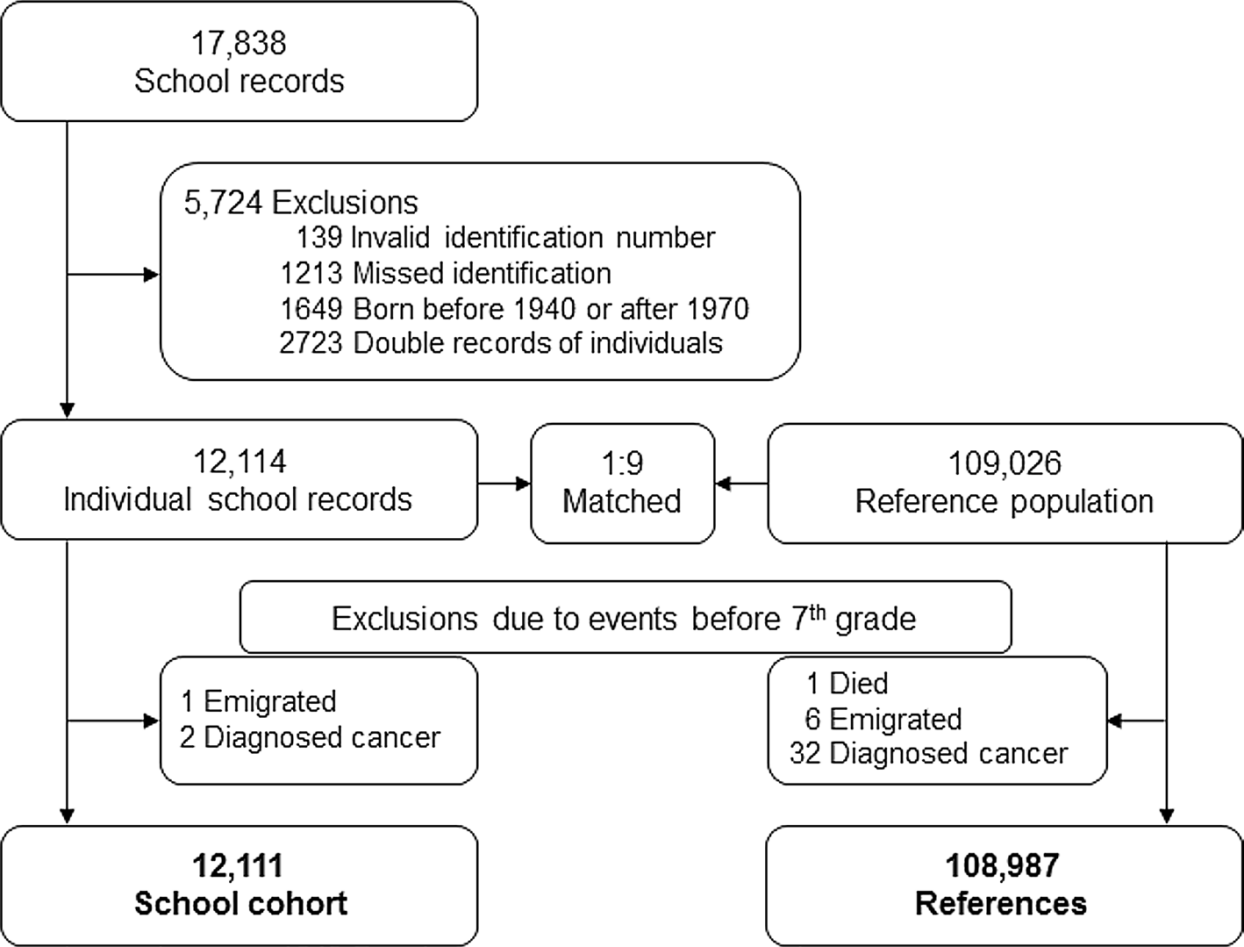
A flow chart describing the study population selection

School records were obtained from the Aalborg City Archives in the form of historical handwritten files, N = 17,838 (Fig. 2). The only school records saved in the archive were from seventh grades. We restricted the birth cohort to the 1940–1970 period for three reasons: to be able to identify participants in relevant national registers, to ensure a long follow-up period (end of 2015) and to include only pupils who attended the schools before the Danish asbestos ban in 1986. We excluded 5724 school records due to lack of identification, birthdate outside the cohort period or duplicate records (Fig. 2). The school cohort was categorised by gender and 5-year birth periods; and an age and sex-matched 1:9 reference cohort was established by randomly selecting participants using the unique personal identification number (CPR) in the Danish Civil Registration System. Inclusion was restricted to participants who up to the beginning of seventh grade (age 12) were still alive and had not been diagnosed with any cancer, equalling the available school records. The final cohort included 12,111 pupils from the four schools in Aalborg and 108,987 reference participants from all over Denmark who had not attended these schools (Fig. 2).

### Registers

Data for this retrospective study up to the end of 2015 were obtained by linkage of records from three national Danish registers. First, we used the Danish Civil Registration System to identify individuals from the school cohort and later the matched reference cohort described above. The Danish Civil Registration System was established in 1968. In this system, all residents in Denmark are registered and assigned a unique CPR number. The register is continuously updated, holding, among others, information on sex, birth date and birth parish, disappearance, emigration and death; and information on parents, spouse and any children. Most of the school records were not assigned a CPR number as the records were written before 1968. However, information about name, birth date and birth parish was used to link record holders’ identities to the CPR assigned to them in 1968. Unfortunately, this linkage was impossible for 1213 subjects (Fig. 2). Using the CPR number, we could establish accurate data linkage to the two other registers used in the study at the individual level [12]. Second, information of outcome was extracted from the Danish Cancer Registry, which was established in 1943 [13]. This register keeps information about dates of diagnosis of all primary cancers classified according to the International Classification of Diseases (ICD). We used the ICD-7 (1943–1977), the ICD-O (1978–2003) and the ICD-10 (2004 onwards). All ICD-7 and ICD-O codes were revised into ICD-10 codes by clinical expert review. To avoid possible underestimation due to survivor bias, we started follow-up on haematological malignancies on 2 April 1968, i.e. on the date at which all residents in Denmark received a CPR number. Thirdly, information on individual employment history was extracted from the Supplementary Pension Fund Register (ATP) in order to assess all participating individuals’ occupational asbestos exposure. This register keeps data on all employments, including dates of employment and discontinuation within a company backdating to 1964 for all wage earners aged 16–66 working ≥ 9 h/week. Companies are classified based on a 5-digit extended Danish version of the UN industry classification, ISIC Rev. 2 (DSE77) [14]. The ATP does not include information on the job performed in the company or any self-employments.

### Haematological malignancies

Haematological malignancy was restricted to first recorded primary cancer, excluding non-melanoma skin cancers. Haematological malignancies were identified for eight subgroups defined by their ICD-10 codes: (1) Hodgkin lymphoma (C81); (2) Non-Hodgkin lymphoma and malignant immunoproliferative disease (C82–85, C88.3–88.9); (3) Multiple myeloma (C90, C88.0–88.2); (4) Lymphatic leukaemia (C91); (5) Myeloid leukaemia (C92); (6) Monocytic leukaemia (C93); (7) Other and unspecified leukaemia (C94-95); (8) Other and unspecified cancer in lymphatic and haematopoietic tissue (C96). Most of the subgroups included few cases in the school cohort. Analyses were restricted to overall haematological malignancy (1–8) and the following combined subgroups: lymphoma (Hodgkin, non-Hodgkin, and multiple myeloma (1–3)) and leukaemia (lymphatic leukaemia, myeloid leukaemia, monocytic leukaemia, and other leukaemia (4–7)).

### Assessment of occupational exposure

To distinguish the effect of childhood environmental asbestos exposure from the effect of occupational asbestos exposure occurring in adulthood, occupational asbestos exposure was addressed as a possible confounder. Childhood environmental exposure/neighbourhood was analysed in relation to adulthood neighbourhood and in relation to occupational asbestos exposure. We used the Danish version of the related JEM from the Nordic Occupational Cancer Study (NOCCA) to trace employment history prior to haematological malignancy [21]. The Danish version included 32 codes for professions in which workers were potentially exposed to occupational asbestos. The total number of codes included in the Danish industry codes is 579 (DSE77). The NOCCA JEM was based on available data and expert opinions. The NOCCA JEM includes two metrics for each job: a) the prevalence (%) of exposed workers and b) the level of exposure (asbestos: f/cm^3^). These metrics were available for three specific calendar periods (1960–1974, 1975–1984, 1985–1994). We used two different JEM models: First, the prevalence of exposed individuals (a) was used to dichotomize the ATP trade codes into a variable of occupational exposure (JEM1 model: asbestos > 50%). This cut-off was set to reflect the estimated proportion of occupational exposed Danes of 10% among the controls [22]. Second, we made a JEM model using a multiplication of prevalence and level (a*b) of exposure. An experienced specialist in occupational medicine (ØO) assessed the JEM2 model cut-off, ≥ 0.1. Jobs included in both JEMs were jobs at, e.g., “iron shipyards”, “building and carpentry firms” and the DEF. Jobs only included in JEM1 were, e.g., “firefighters”. Jobs only included in JEM2 were “stove installation” and various jobs within the category “general contracting businesses”. The cumulative exposure was assessed by years within multiple lifetime-recorded eligible jobs. Some dose-response trends are often identified in occupational job exposures; thus, additional analyses were performed with exposure for more than 10 years (> 10 year) and more than 15 years (> 15 year) in both JEM models. The final JEM models were then: JEM1, JEM1 > 10 year, JEM1 > 15 year, JEM2, JEM2 > 10 year and JEM2 > 15 year.

### Statistics

Age was used as time scale for all analyses. Thus, individuals of similar age were compared. Standardised incidence ratios (SIRs) were estimated according to sex, years in study and calendar time. Both years in study and calendar time were calculated with 5-year intervals. For test between subgroups, chi-squared was used for categorical variables and Wilcoxon’s rank-sum test for non-normally distributed continuous variables. The significance level was set at 5% for all tests. Statistical analyses were conducted in Stata 15.1 (StataCorp LLC, 2017). Cox regression was used to estimate cause-specific hazard ratios (HRs) for haematological malignancy as primary cancer, thus distinguishing events of interest from competing events. Competing events were death or other cancer types except skin cancer, which excluded occurrence of haematological malignancy as primary cancer. The crude cumulative incidence rate for occurrence of primary haematological malignancy was estimated separately for exposed and unexposed individuals using the Nelson-Aalen estimator. Individuals experiencing competing risks were considered at risk only until event. Participants were censored at the date of emigration or loss to follow-up. Cox regression was performed for all haematological malignancies as primary analysis and as secondary analysis for lymphoma and leukaemia separately. All regressions included the following covariates in addition to the exposure of interest (indicator variable for attending seventh grade at one of the four schools near to and downwind of the DEF): calendar time, sex and occupational exposure to asbestos. Occupational exposure was dichotomized as described above into two models (JEM1 and JEM2). The assumptions of proportional HR in Cox regression were investigated both with a rank-sum test for proportionality and by exploration of the Schoenfeld residuals.

SIR sensitivity analyses included a 10-year lag time of events. In the cause-specific HR, six sensitivity analyses were performed to consider different aspects of the study. First, the analysis was restricted to participants born in Denmark. This was done since Denmark has no recognised naturally occurring asbestos [23], and unknown natural asbestos exposure could be reduced by restricting the analysis to participants born in Denmark. Second, the analysis was restricted to schoolchildren born in parishes within a range of three kilometres from the DEF, combined with a reference cohort born in Denmark but outside these parishes. Third, the analysis was performed on participants born after January 1946 to ensure a possible uninterrupted environmental exposure as the DEF could not import asbestos during World War II. Furthermore, in 1964 these participants were 18 years old and expected to have a full employment history in the ATP (established in April 1964). In the fourth analysis, the missing occupational exposure among self-employed was analysed by assigning these participants to have no occupational exposure to asbestos; and in the fifth analysis, all to have an occupational asbestos exposure. The sixth analysis restricted the schoolchildren to the two schools closest to the DEF. All sensitivity analyses were conducted for all JEM1 and JEM2 models. Furthermore, to explain away the observed exposure-outcome association, the E-value was used to assess the level of relative effect of an unmeasured confounder, with a relative effect measure with both outcome and exposure, conditioned on the measured covariates [24].

## Results

Table 1 lists the characteristics of the cohortees who were nearly equally divided between females and males. There were more Danish native-born participants in the school cohort than in the reference cohort. By far the most common haematological malignancy was non-Hodgkin lymphoma. Participants in the school cohort experienced higher occupational asbestos exposure than reference cohort participants.

**Table 1:** Characteristics of the study participants stratified by school cohort and reference cohort at the end of follow-up, N = 121,098

Variable, n (%)	School cohort		Reference cohort		*p* value
Sex	12,111	(10.0)	108,987	(90.0)	
Female	6024	(49.7)	54,200	(49.7)	
Male	6087	(50.3)	54,787	(50.3)	0.99
Age, median (range)	*62.4*	*(13.0*–*76.0)*	*62.1*	*(12.0*–*76.0)*	< 0.01
Follow-up, median (range)	*49.4*	*(1.2*–*63.4)*	*49.0*	*(0.1*–*63.4)*	< 0.01
Born in Denmark^a^	11,834	(99.1)	95,098	(87.8)	< 0.01
Death	1898	(15.7)	15,081	(13.8)	< 0.01
Overall haematological malignancies	110	(0.9)	1,015	(0.9)	0.80
Hodgkin lymphoma (C81)	14	(0.1)	96	(0.1)	0.34
Non-Hodgkin lymphoma and malignant immunoproliferative disease (C82–85, C88.3–88.9)	45	(0.4)	450	(0.4)	0.50
Multiple myeloma (C90, C88.0–88.2)	8	(0.1)	152	(0.1)	0.04
Lymphatic leukaemia (C91)	18	(0.2)	166	(0.2)	0.92
Myeloid leukaemia (C92)	19	(0.2)	134	(0.1)	0.32
Monocytic leukaemia (C93)	3	(0.02)	6	(0.01)	0.02
Other and unspecified leukaemia (C94–95)	1	(0.01)	8	(0.01)	0.91
Other and unspecified cancer in lymphatic and haematopoietic tissue (C96)	2	(0.02)	3	(< 0.01)	0.03
Wage earning years, median (range)^b^	*27.9*	*(0.02*–*58.2)*	*28.7*	*(0.02*–*78.2)*	< 0.01
Occupational asbestos exposure^b^					
No	9777	(82.7)	91,501	(89.4)	
Yes; JEM1	2048	(17.3)	10,888	(10.6)	< 0.01

^a^Missing information: school n = 164, reference n = 625

^b^Non-wage earners: school n = 286, reference n = 6598, Job exposure matrix model: JEM1 based on prevalence of exposed employees

The SIR provided no evidence for a difference between the cohorts for haematological malignancy (SIR 0.94, 95% CI 0.78–1.13), combined groups of lymphoma (SIR 0.83, 95% CI 0.65–1.05) or leukaemia (SIR 1.13, 95% CI 0.83–1.53). Including a 10-year lag period in the SIR calculation did not change these results.

The two JEM models are compared in Table 2. Generally, there were more exposed participants when using the JEM1 model to define the exposure prevalence. Although differences between JEM1 and JEM2 were large, they were not statistically significant, *p* = 0.06. However, the difference between JEM1 and JEM2 was clearly statistically significant when occupational asbestos exposure was extended to 10 and 15 years.

**Table 2:** Comparing the groups of exposed and unexposed for occupational asbestos in the two JEM models

	JEM2^b^			*p* value
	Unexposed	Exposed	Total	
JEM1^a^				
Unexposed	96,856	4422	101,278	
Exposed	4599	8337	12,936	
Total	101,455	12,759	114,214	0.06

	JEM2 > 10			
	Unexposed	Exposed	Total	
JEM1 > 10				
Unexposed	111,788	448	112,236	
Exposed	1287	691	1978	
Total	113,075	1139	114,214	< 0.001

	JEM2 > 15			
	Unexposed	Exposed	Total	
JEM1 > 15				
Unexposed	112,661	252	112,913	
Exposed	960	341	1301	
Total	113,621	593	114,214	< 0.001

^a^JEM1: The exposed occupations defined as those with > 50% prevalence of exposed employees

^b^JEM2: Based on the prevalence of exposed employees * the level (f/cm^3^) of trade exposure, the exposed is defined as ≥ 0.1 in a multiplication of prevalence and level of asbestos exposure

**Fig. 3:**
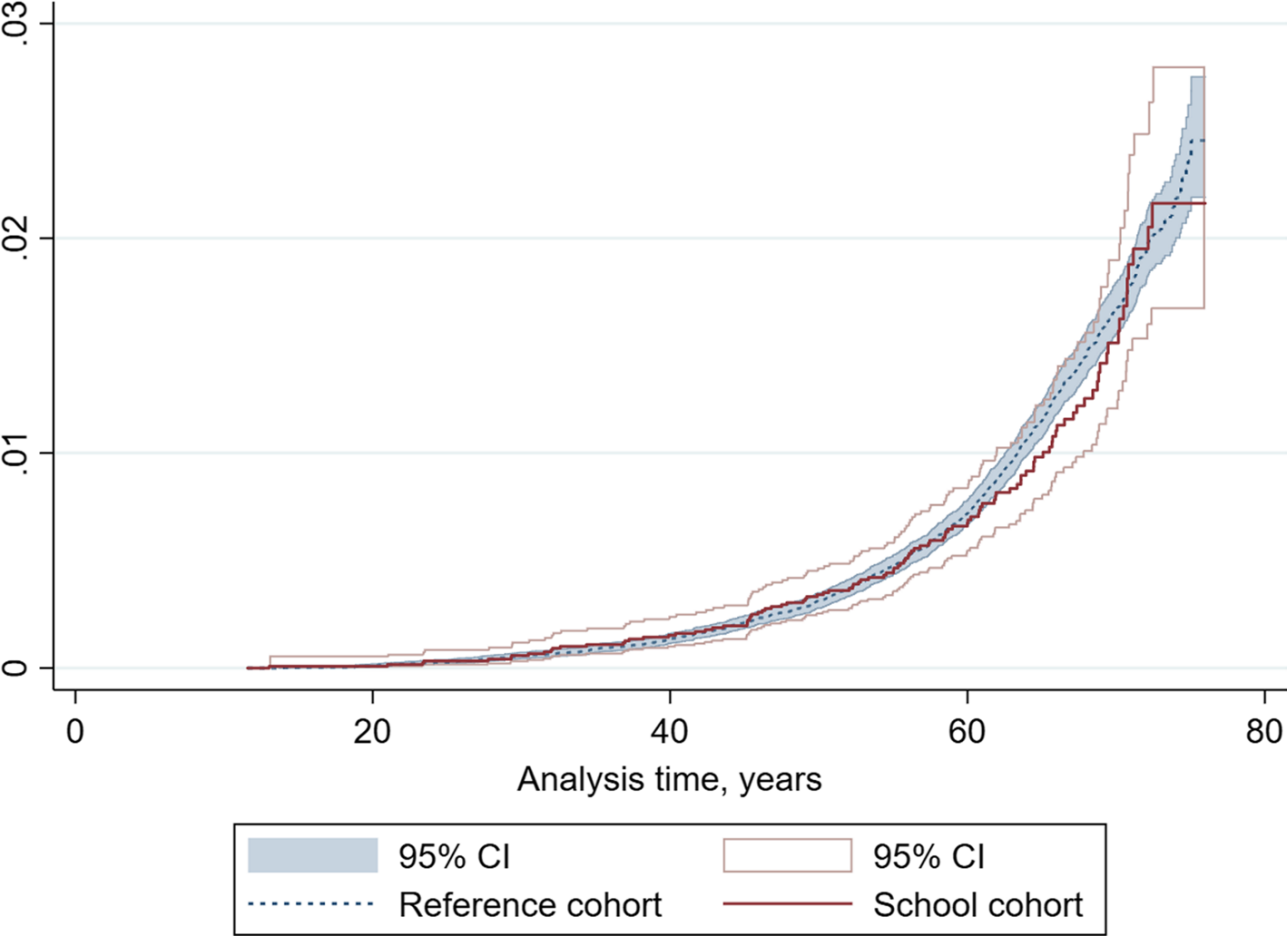
Illustrates the cumulative incidence of haematological malignancy in the cohorts of schoolchildren and references

Estimates for childhood environmental exposure remained similar and essentially null regardless of outcome and regardless of which JEM was used for occupational exposures (Tables 3, 4). Moreover, the crude cause-specific hazard ratio estimates were similar to the adjusted estimates, for all outcomes, among the schoolchildren close to the DEF. Figure 3 illustrates the cumulative incidence of haematological malignancy in the cohorts of schoolchildren and references. Cox analyses including the occupational exposure yielded a total of 5,440,823 person years at risk. Calendar time did not influence the estimates and was not included in the final models. Table 3 shows the HRs using the JEM1 model for all outcomes and times of occupational asbestos exposure. For leukaemia, the 10-year occupational asbestos exposure shows an increased risk, JEM1 > 10 year.

**Table 3:** Cox regression of three haematological malignancy outcomes of first cancers, with other cancers and death as competing risks, in a job exposure matrix model based on the prevalence of exposure (JEM1) to occupational asbestos, n = 114,214

	JEM1			JEM1 > 10 year			JEM1 > 15 year		
	Cases	HR	(95% CI)	Cases	HR	(95% CI)	Cases	HR	(95% CI)
Haematological malignancy									
Population									
Reference	989	1		989	1		989	1	
School	106	0.92	(0.75–1.13)	106	0.92	(0.75–1.12)	106	0.92	(0.75–1.12)
Sex									
Female	430	1		430	1		430	1	
Male	665	1.58	(1.39–1.79)	665	1.56	(1.38–1.76)	665	1.57	(1.39–1.77)
Asbestos exposure									
No	935	1		1,061	1		1,073	1	
Yes	160	0.98	(0.82–1.17)	34	1.21	(0.86–1.71)	22	1.16	(0.76–1.77)
Combined lymphoma									
Population									
Reference	683	1		683	1		683	1	
School	66	0.83	(0.65–1.07)	66	0.83	(0.64–1.07)	66	0.83	(0.64–1.07)
Sex									
Female	288	1		288	1		288	1	
Male	461	1.65	(1.42–1.92)	461	1.63	(1.41–1.89)	461	1.63	(1.40–1.89)
Asbestos exposure									
No	643	1		730	1		736	1	
Yes	106	0.94	(0.76–1.16)	19	0.98	(0.62–1.55)	13	0.99	(0.57–1.72)
Combined leukaemia									
Population									
Reference	303	1		303	1		303	1	
School	38	1.07	(0.76–1.50)	38	1.06	(0.76–1.49)	38	1.07	(0.76–1.50)
Sex									
Female	140	1		140	1		140	1	
Male	201	1.44	(1.15–1.80)	201	1.42	(1.14–1.77)	201	1.44	(1.16–1.79)
Asbestos exposure									
No	288	1		326	1		332	1	
Yes	53	1.07	(0.79–1.46)	15	1.78	(1.05–3.00)	9	1.56	(0.80–3.04)

Missing information for non-wage earners: School n = 286, reference n = 6598

*HR* hazard ratio,* CI* confidence interval

**Table 4:** Cox regression of three haematological malignancy outcomes of first cancers, with other cancers and death as competing-risks, in a job exposure matrix model based on prevalence of exposed employees * the level of trade exposure (JEM2) for occupational asbestos, n = 114,214

	JEM2			JEM2 > 10 year			JEM2 > 15 year		
	Cases	HR	(95% CI)	Cases	HR	(95% CI)	Cases	HR	(95% CI)
Haematological malignancy									
Population									
Reference	989	1		989	1		989	1	
School	106	0.92	(0.75–1.12)	106	0.91	(0.75–1.11)	106	0.91	(0.75–1.12)
Sex									
Female	430	1		430	1		430	1	
Male	665	1.56	(1.37–1.77)	665	1.56	(1.38–1.76)	665	1.56	(1.38–1.76)
Asbestos exposure									
No	917	1		1,068	1		1,078	1	
Yes	178	1.05	(0.88–1.24)	27	1.47	(1.00–2.16)	17	1.69	(1.04–2.73)
Combined lymphoma									
Population									
Reference	683	1		683	1		683	1	
School	66	0.83	(0.64–1.07)	66	0.83	(0.64–1.07)	66	0.83	(0.64–1.07)
Sex									
Female	288	1		288	1		288	1	
Male	461	1.63	(1.40–1.90)	461	1.62	(1.40–1.88)	461	1.62	(1.40–1.88)
Asbestos exposure									
No	632	1		734	1		740	1	
Yes	117	0.99	(0.80–1.22)	15	1.18	(0.71–1.98)	9	1.30	(0.67–2.51)
Combined leukaemia									
Population									
Reference	303	1		303	1		303	1	
School	38	1.06	(0.76–1.49)	38	1.05	(0.75–1.48)	38	1.06	(0.76–1.48)
Sex									
Female	140	1		140	1		140	1	
Male	201	1.41	(1.12–1.77)	201	1.42	(1.14–1.77)	201	1.43	(1.15–1.78)
Asbestos exposure									
No	281	1		329	1		333	1	
Yes	60	1.18	(0.88–1.59)	12	2.14	(1.19–3.84)	8	2.59	(1.28–5.26)

Missing information for non-wage earners: School n = 286, reference n = 6598.

*HR* hazard ratio,* CI* confidence interval

Using the other JEM2 model in the analyses (Table 4), we found, a slightly stronger impact of the long occupational asbestos exposures for overall haematological malignancy (JEM2 > 15 year) and for leukaemia (JEM2 > 10 year and JEM2 > 15 year). However, the numbers of exposed cases were quite small in both JEM models when we looked at the long-term occupational asbestos exposure. Males had a significantly increased risk of haematological malignancy in all the investigated outcomes, regardless of JEM model and time of occupational exposure, HR range 1.41–1.65. No interaction was identified between environmental and occupational asbestos exposure in any of the models. The estimates from most of the sensitivity analyses were similar to those presented in Tables 3, 4 (Online Resource 1–3). However, restricting the analysis to participants born after January 1946 (n = 85,614), when importation of asbestos began to increase rapidly, we found higher estimates for occupational asbestos exposure in both JEM models and all exposure time levels for all outcomes (e.g. haematological malignancy: JEM2 > 10 year, HR 2.58, 95% CI 1.57–4.26 rather than 1.47, 95% CI 1.00–2.16). Restricting the analysis to include schoolchildren only from the two school closets to the DEF slightly increased the risk range of environmental exposure for leukaemia, HR range 1.05–1.07 to 1.31–1.33, without any significance. We calculated the E-value of the smallest significant leukaemia estimate from Table 3–4: model JEM1 > 10 year to HR 2.96, 95% CI 1.28–5.45.

## Discussion

This study adds new insights regarding environmental and occupational asbestos exposure and haematological malignancy. Environmental asbestos exposure in childhood was not confirmed as a risk factor for haematological malignancy in this study. However, the study suggests that long-term occupational asbestos exposure is associated with a risk for haematological malignancy, notably for leukaemia.

Only little prior research has been devoted to the study of the association between asbestos exposure in childhood and the risk of haematological cancers. Reid et al. conducted a study of 2,460 children exposed solely to environmental asbestos in Wittenoom, Australia, with a mean follow-up age of 42.4 years. They observed an increased risk for leukaemia (n = 7, not defined in details) in males (SIR 4.62, 95% CI 1.86–9.53); adjusted for smoking, the SIR was 2.66, 95% CI 1.07–5.47 [25]. Our analysis does not support those results, SIR 1.13, 95% CI 0.83–1.53 for leukaemia. This disparity may be due to our larger cohort, our slightly longer follow-up or the fact that the environmental asbestos exposure in our cohort was mainly chrysotile, while Reid's population was exposed mainly to crocidolite. Although our cohort was larger, our data on time of asbestos exposure were less accurate than those Reid et al. presented. Furthermore, our 10-year latency SIR did not change these estimates.

Regarding the occupational asbestos exposure, two recent reviews of haematological malignancy and occupational asbestos exposure arrived at partially opposite conclusions. The systematic review by Becker et al. indicated that occupational asbestos exposure was associated with a weakly increased risk for non-Hodgkin lymphoma and chronic lymphatic leukaemia but not multiple myeloma [7]. They included 13 studies of non-Hodgkin lymphoma, three studies of chronic lymphatic leukaemia and ten studies of multiple myeloma, mostly case-control studies with less than 100 highly exposed cases and no time span of exposure. No quality criteria for inclusion of studies was displayed, and only explorative estimates were offered. In contrast, the review by Weisenburger et al. concluded that there was no association between haematolymphoid cancers and occupational asbestos exposure [8]. They included 35 epidemiological studies (16 studies also included in the study by Becker et al.) and found 32 with no association. For the outcome of lymphoma, we found a non-significant association for occupational asbestos exposure, but our results suggest an increased risk for leukaemia after long-term occupational asbestos exposure.

### Strengths and weaknesses

Our data originate from objective and validated Danish registers and include a large population-based cohort with accurate and unique identification of each study participant. The follow-up period was long, and we were able to differentiate between environmental exposure and occupational exposure due to the well-delimitated location of the defined school cohort and a comprehensive employment history from the ATP. Furthermore, the outcome originates from the validated Danish Cancer Registry, which has more than 75 years of experience in nationwide cancer registration [13]. Moreover, the subgroup analyses seem important in this context because they highlight differences between lymphomas and leukaemia, especially among participants born after World War II.

Most likely, the environmental asbestos exposure is misclassified or at least not differentiated. Only school records from the seventh grade were available. We do not know if the children went to the school for one or maybe seven years, and we do not know whether they lived all their childhood near the DEF. Thus, we have used a very coarse way to divide the environmental asbestos exposure without any time of exposure. This approach will probably underestimate the association when less exposed children are included in line with the children who lived near the DEF for their entire childhood. Furthermore, the various distances of the four schools to the DEF would also affect the environmental asbestos exposure. In the sixth sensitivity analysis, we saw a slight increase in the risk for leukaemia in children attending the two closest schools, probably due to a larger environmental exposure. The environmental exposure has been verified in another paper using the same cohort. In this study, the school cohort experienced a high, increased risk of mesothelioma compared with the reference cohort, HR 7.15, 95% CI 4.54–11.27, when adjusted for occupational and domestic asbestos exposure [19]. The domestic exposure (e.g. father working at DEF) was not included in this study as the initial anticipation was that this exposure would not affect the children to any noticeable extent. However, the domestic covariate would have been included if the study had more power.

ATP membership is limited to wage earners and does not include self-employments, which might introduce a non-differential underestimation of exposure. The fourth sensitivity analysis allocated those without ATP data into no exposure, with similar results. In the fifth sensitivity analysis, participants with missing ATP data were analysed as participants who were occupationally exposed. We found a slightly increased risk estimate only for leukaemia. The identification of occupational exposure at the company level with a mixed identification of white-collar and blue-collar workers creates a substantial non-differential misclassification. This might tend to underestimate the risk as too many will be classified as exposed. On the other hand, the use of ATP data excludes recall bias. The different JEM models yield significant risk estimates for leukaemia for those who had been working in the industry for more than 10 years. Though the models were significantly different, this might underline the difficulty and uncertainty in using JEM estimation of exposure, but it strengthens the confidence of an association with leukaemia. Despite the relatively few cases with haematological malignancies and the sometimes wide CIs in the present study, we assume that use of the JEMs with long-term records of occupational exposure outperforms simple never/ever dichotomization for exposure assessment in a manner similar to that of a dose-response effect. A healthy worker effect, with those who stay healthy working longer in the exposed trades, and the low number of cases in the population are also important contributors to the moderate results in the present study. Thus, the study lacked power to stratify further on solely environmental, solely occupational and combined environmental and occupational exposure, which could otherwise have contributed to a better differentiation of the estimates. Robins’ generalized methods (g methods) could provide more consistent estimates under a less restrictive set of identification conditions than the Cox regression [26]. This would be too comprehensive to include in this paper, and is suggested as an issue for further investigation.

Other potential confounders could be benzene, radiation or pesticide exposure. However, we have no reason to think that these variables would also be associated with the schoolchildren’s proximity to the DEF. The association between occupational asbestos exposure and leukaemia might be skewed due to other socio-economic factors or missing tobacco information. We selected a huge reference cohort to reduce the socio-economic parameter. Matching the school cohort to another reference school cohort would have been another strategy to consider. However, this strategy was regarded as very comprehensive with few advantages compared to the large register cohort. Tobacco smoking is associated with myeloid leukaemia with an estimated incidence rate ratio of 1.5 [27, 28]. Myeloid leukaemia accounts for 43% of the leukaemia group in our study, and the association between asbestos exposure and disease might therefore be overestimated. We assume that both cohorts have a high prevalence of smoking. However, a substantial difference in smoking is needed to establish any confounder effect as the relative risk of smoking is relatively low. Since calendar time influences occupational exposure, and might influence the outcome as well, we included calendar time in the regression model. However, calendar time did not influence the results. Furthermore, calendar time is taken into account in the JEM models, with different exposures related to different calendar periods. Calendar time did not influence the estimates and was not included in the final models. The estimated E-value depicts that an unmeasured confounder (e.g. smoking) should have a relatively high effect level with both outcome and exposure to explain the observed exposure-outcome association estimated for leukaemia JEM1 > 10 year: HR 2.96, 95% CI 1.28–5.45. Given that no other unmeasured confounders are likely, a causal interpretation of an occupational asbestos effect might be straightforward if the present results can be verified in other studies. Biological plausibility for this relationship is supported by the fact that asbestos fibres have been found in bone marrow, verifying that asbestos bodies may be found beyond the respiratory system [2]. The pathophysiological mechanism of this is unknown, but it may mirror mechanisms seen for malignant mesothelioma, viz cytotoxicity, inflammation, changed epigenetics and abnormal segregation at mitosis [11]. Occupationally asbestos exposure cannot be neglected as a potential risk factor for leukaemia.

## Conclusion

Our study does not support an association between environmental asbestos exposure in childhood and haematological malignancies. However, long-term occupational asbestos exposure may be a risk factor for leukaemia. Countries still using asbestos should acknowledge this additional cancer risk in their asbestos management. Additionally, all countries that ever used asbestos ought to be aware of this additional cancer risk in future handling of asbestos material, especially outranged and peeling materials. Furthermore, it should be considered to revise national compensation regulations if the present results can be verified in other studies.

## Electronic supplementary material

Below is the link to the electronic supplementary material.

Supplementary material 1 (PDF 427 kb)
