# Supplementary material for: Asbestos exposure and haematological malignancies: a Danish cohort study
Source: Eur J Epidemiol. 2020 Feb 10;35(10):949–60. doi: 10.1007/s10654-020-00609-4 (PMC7524705; doi:10.1007/s10654-020-00609-4)
Supplement: Supplementary file 1 — Supplementary material 1 (PDF 427 kb) [file 10654_2020_609_MOESM1_ESM.pdf]

European Journal of Epidemiology

Title

Asbestos exposure and haematological malignancies – a Danish cohort study

Authors

Else Toft Würtz[1], Johnni Hansen[2], Oluf Dimitri Røe[3-4], Øyvind Omland[1,5].

Affiliations

[1]Department of Occupational and Environmental Medicine, Danish Ramazzini Centre, Aalborg University Hospital, DK-9000 Aalborg, Denmark, [2]Danish Cancer Society Research Center, DK-2100 Copenhagen Ø, Denmark, [3]Department of Clinical Research and Molecular Medicine, Norwegian University of Science and Technology, N-7491 Trondheim, Norway, [4]Clinical Cancer Research Center, Department of Clinical Medicine, Aalborg University Hospital, DK-9000 Aalborg, Denmark, [5]Department of Clinical Medicine, Aalborg University, DK-9000 Aalborg, Denmark

Correspondence to: Else Toft Würtz etw@rn.dk

Department of Occupational and Environmental Medicine, Aalborg University Hospital

Havrevangen 1, DK-9000 Aalborg, Denmark

SENSITIVITY ANALYSES

In the cause-specific HR, six sensitivity analyses were performed to consider different aspects of the study. First, the analysis was restricted to participants born in Denmark. This was done since Denmark has no recognised naturally occurring asbestos [23], and unknown natural asbestos exposure could be reduced by restricting the analysis to participants born in Denmark. Second, the analysis was restricted to schoolchildren born in parishes within a range of three kilometres from the DEF, combined with a reference cohort born in Denmark but outside these parishes. Third, the analysis was performed on participants born after January 1946 to ensure a possible uninterrupted environmental exposure as the DEF could not import asbestos during World War II. Furthermore, in 1964 these participants were 18 years old and expected to have a full employment history in the ATP (established in April 1964). In the fourth analysis, the missing occupational exposure among self-employed was analysed by assigning these participants to have no occupational exposure to asbestos; and in the fifth analysis, all to have an occupational asbestos exposure. The sixth analysis restricted the schoolchildren to the two schools closest to the DEF. All sensitivity analyses were conducted for all JEM1 and JEM2 models.

Online resource 1: Sensitivity analyses: Cox regression of hematological malignancy as first cancer, with other cancers and death as competing-risk, with different job exposure matrix models for occupational asbestos exposure adjustment.

		From Table 3 or 4			First			Second			Third			Fourth			Fifth			Sixth		
n		114,214			104,301			99,780			85,614			121,098			121,098			108,217		
					Born in Denmark (DK)			Schoolchildren born in Aalborg, others in DK			Born after January 1946			ATP missing into no asbestos exposure			ATP missing into asbestos exposure			Children from the two schools closets to DEF		
JEM1		Cases	HR	(95% CI)	Cases	HR	(95% CI)	Cases	HR	(95% CI)	Cases	HR	(95% CI)	Cases	HR	(95% CI)	Cases	HR	(95% CI)	Cases	HR	(95% CI)
Population																						
	Reference	989	1		936	1		919	1		587	1		1015	1		1015	1		989	1	
	School	106	0.92	(0.75-1.13)	105	0.92	(0.75-1.12)	76	0.88	(0.70-1.12)	62	0.87	(0.67-1.13)	110	0.94	(0.77-1.15)	110	0.94	(0.77-1.14)	57	0.93	(0.71-1.22)
Sex																						
	Female	430	1		413	1		395	1		268	1		446	1		446	1		412	1	
	Male	665	1.58	(1.39-1.79)	628	1.56	(1.37-1.78)	600	1.56	(1.37-1.78)	381	1.42	(1.21-1.67)	679	1.56	(1.38-1.76)	679	1.54	(1.36-1.74)	634	1.56	(1.37-1.77)
Asbestos exposure																						
	No	935	1		884	1		847	1		554	1		965	1		935	1		894	1	
	Yes	160	0.98	(0.82-1.17)	157	0.99	(0.83-1.18)	148	0.98	(0.82-1.18)	95	1.09	(0.87-1.37)	160	0.98	(0.82-1.16)	190	1.04	(0.88-1.22)	152	1.01	(0.84-1.20)
JEM1 >10 years																						
Population																						
	Reference	989	1		936	1		919	1		587	1		1015	1		1015	1		989	1	
	School	106	0.92	(0.75-1.12)	105	0.91	(0.75-1.12)	76	0.88	(0.69-1.11)	62	0.87	(0.67-1.13)	110	0.94	(0.77-1.14)	110	0.94	(0.77-1.14)	57	0.93	(0.71-1.22)
Sex																						
	Female	430	1		413	1		395	1		268	1		446	1		446	1		412	1	
	Male	665	1.56	(1.38-1.76)	628	1.54	(1.36-1.75)	600	1.54	(1.36-1.75)	381	1.42	(1.22-1.67)	679	1.54	(1.37-1.74)	679	1.54	(1.36-1.73)	634	1.55	(1.36-1.75)
Asbestos exposure																						
	No	1061	1		1007	1		962	1		629	1		1091	1		1061	1		1013	1	
	Yes	34	1.21	(0.86-1.71)	34	1.24	(0.88-1.75)	33	1.27	(0.89-1.80)	20	1.59	(1.01-2.48)	34	1.21	(0.86-1.70)	64	1.31	(1.02-1.69)	33	1.28	(0.90-1.82)
JEM1 >15 years																						
Population																						
	Reference	989	1		936	1		919	1		587	1		1015	1		1015	1		989	1	
	School	106	0.92	(0.75-1.12)	105	0.92	(0.75-1.12)	76	0.88	(0.70-1.11)	62	0.87	(0.67-1.14)	110	0.94	(0.77-1.14)	110	0.94	(0.77-1.14)	57	0.93	(0.71-1.22)
Sex																						
	Female	430	1		413	1		395	1		268	1		446	1		446	1		412	1	
	Male	665	1.57	(1.39-1.77)	628	1.55	(1.37-1.76)	600	1.55	(1.36-1.76)	381	1.43	(1.22-1.67)	679	1.55	(1.37-1.74)	679	1.54	(1.37-1.74)	634	1.55	(1.37-1.76)
Asbestos exposure																						
	No	1073	1		1019	1		973	1		634	1		1103	1		1073	1		1025	1	
	Yes	22	1.16	(0.76-1.77)	22	1.18	(0.77-1.80)	22	1.23	(0.81-1.89)	15	1.79	(1.07-3.00)	22	1.15	(0.75-1.76)	52	1.31	(0.99-1.73)	21	1.19	(0.77-1.84)
JEM2																						
Population																						
	Reference	989	1		936	1		919	1		587	1		1015	1		1015	1		989	1	
	School	106	0.92	(0.75-1.12)	105	0.91	(0.75-1.12)	76	0.88	(0.69-1.11)	62	0.86	(0.66-1.12)	110	0.94	(0.77-1.14)	110	0.93	(0.77-1.14)	57	0.93	(0.71-1.22)
Sex																						
	Female	430	1		413	1		395	1		268	1		446	1		446	1		412	1	
	Male	665	1.56	(1.37-1.77)	628	1.54	(1.35-1.75)	600	1.55	(1.36-1.77)	381	1.40	(1.19-1.65)	679	1.54	(1.36-1.74)	679	1.52	(1.34-1.72)	634	1.54	(1.36-1.76)
Asbestos exposure																						
	No	917	1		867	1		833	1		550	1		947	1		917	1		878	1	
	Yes	178	1.05	(0.88-1.24)	174	1.05	(0.89-1.25)	162	1.03	(0.86-1.23)	99	1.18	(0.94-1.47)	178	1.04	(0.88-1.23)	208	1.10	(0.94-1.29)	168	1.06	(0.89-1.26)
JEM2 >10 years																						
Population																						
	Reference	989	1		936	1		919	1		587	1		1015	1		1015	1		989	1	
	School	106	0.91	(0.75-1.11)	105	0.91	(0.74-1.11)	76	0.87	(0.69-1.10)	62	0.86	(0.66-1.12)	110	0.93	(0.76-1.13)	110	0.94	(0.77-1.14)	57	0.93	(0.71-1.21)
Sex																						
	Female	430	1		413	1		395	1		268	1		446	1		446	1		412	1	
	Male	665	1.56	(1.38-1.76)	628	1.54	(1.36-1.74)	600	1.54	(1.35-1.75)	381	1.42	(1.21-1.66)	679	1.54	(1.36-1.73)	679	1.54	(1.36-1.73)	634	1.54	(1.36-1.74)
Asbestos exposure																						
	No	1068	1		1014	1		970	1		633	1		1098	1		1068	1		1019	1	
	Yes	27	1.47	(1.00-2.16)	27	1.50	(1.02-2.21)	25	1.47	(0.99-2.20)	16	2.58	(1.57-4.26)	27	1.46	(0.99-2.14)	57	1.45	(1.11-1.90)	27	1.62	(1.10-2.39)
JEM2 >15 years																						
Population																						
	Reference	989	1		936	1		919	1		587	1		1015	1		1015	1		989	1	
	School	106	0.91	(0.75-1.12)	105	0.91	(0.74-1.12)	76	0.87	(0.69-1.10)	62	0.87	(0.67-1.13)	110	0.93	(0.77-1.14)	110	0.94	(0.77-1.14)	57	0.93	(0.71-1.21)
Sex																						
	Female	430	1		413	1		395	1		268	1		446	1		446	1		412	1	
	Male	665	1.56	(1.38-1.76)	628	1.54	(1.36-1.75)	600	1.54	(1.36-1.75)	381	1.42	(1.21-1.66)	679	1.54	(1.36-1.73)	679	1.54	(1.37-1.74)	634	1.54	(1.36-1.75)
Asbestos exposure																						
	No	1078	1		1024	1		979	1		637	1		1108	1		1078	1		1029	1	
	Yes	17	1.69	(1.04-2.73)	17	1.72	(1.06-2.78)	16	1.72	(1.05-2.83)	12	3.61	(2.03-6.42)	17	1.68	(1.04-2.72)	47	1.52	(1.13-2.04)	17	1.89	(1.17-3.06)

Online resource 2: Sensitivity analyses: Cox regression of lymphoma as first cancer, with other cancers and death as competing-risk, with different job exposure matrix models for occupational asbestos exposure adjustment.

		From Table 3 or 4			First			Second			Third			Fourth			Fifth			Sixth		
n		114,214			104,301			99,780			85,614			121,098			121,098			108,217		
					Born in Denmark (DK)			Schoolchildren born in			Born after January 1946			ATP missing into no			ATP missing into			Children from the two		
JEM1		Cases	HR	(95% CI)	Cases	HR	(95% CI)	Cases	HR	(95% CI)	Cases	HR	(95% CI)	Cases	HR	(95% CI)	Cases	HR	(95% CI)	Cases	HR	(95% CI)
Population																						
	Reference	683	1		644	1		629	1		411	1		698	1		698	1		683	1	
	School	66	0.83	(0.65-1.07)	66	0.84	(0.65-1.09)	48	0.81	(0.61-1.09)	38	0.77	(0.55-1.07)	67	0.84	(0.65-1.08)	67	0.84	(0.65-1.07)	32	0.76	(0.53-1.09)
Sex																						
	Female	288	1		278	1		264	1		180	1		296	1		296	1		276	1	
	Male	461	1.65	(1.42-1.92)	432	1.61	(1.38-1.88)	413	1.61	(1.38-1.89)	269	1.52	(1.25-1.85)	469	1.63	(1.41-1.90)	469	1.63	(1.40-1.89)	439	1.62	(1.39-1.90)
Asbestos exposure																						
	No	643	1		606	1		577	1		388	1		659	1		643	1		614	1	
	Yes	106	0.94	(0.76-1.16)	104	0.95	(0.77-1.18)	100	0.97	(0.78-1.21)	61	0.99	(0.75-1.30)	106	0.94	(0.76-1.16)	122	0.96	(0.79-1.17)	101	0.97	(0.78-1.20)
JEM1 >10 years																						
Population																						
	Reference	683	1		644	1		629	1		411	1		698	1		698	1		683	1	
	School	66	0.83	(0.64-1.07)	66	0.84	(0.65-1.08)	48	0.81	(0.61-1.09)	38	0.76	(0.55-1.07)	67	0.83	(0.65-1.07)	67	0.83	(0.65-1.07)	32	0.76	(0.53-1.08)
Sex																						
	Female	288	1		278	1		264	1		180	1		296	1		296	1		276	1	
	Male	461	1.63	(1.41-1.89)	432	1.59	(1.37-1.85)	413	1.60	(1.37-1.87)	269	1.51	(1.25-1.83)	469	1.62	(1.40-1.87)	469	1.61	(1.39-1.87)	439	1.61	(1.39-1.88)
Asbestos exposure																						
	No	730	1		691	1		659	1		438	1		746	1		730	1		697	1	
	Yes	19	0.98	(0.62-1.55)	19	1.01	(0.64-1.60)	18	1.00	(0.63-1.61)	11	1.24	(0.68-2.26)	19	0.98	(0.62-1.55)	35	1.03	(0.73-1.44)	18	1.01	(0.63-1.62)
JEM1 >15 years																						
Population																						
	Reference	683	1		644	1		629	1		411	1		698	1		698	1		683	1	
	School	66	0.83	(0.64-1.07)	66	0.84	(0.65-1.08)	48	0.81	(0.60-1.09)	38	0.77	(0.55-1.07)	67	0.83	(0.65-1.07)	67	0.83	(0.65-1.07)	32	0.76	(0.53-1.08)
Sex																						
	Female	288	1		278	1		264	1		180	1		296	1		296	1		276	1	
	Male	461	1.63	(1.40-1.89)	432	1.59	(1.37-1.85)	413	1.60	(1.37-1.87)	269	1.51	(1.25-1.83)	469	1.62	(1.40-1.87)	469	1.61	(1.40-1.87)	439	1.61	(1.39-1.88)
Asbestos exposure																						
	No	736	1		697	1		664	1		442	1		752	1		736	1		703	1	
	Yes	13	0.99	(0.57-1.72)	13	1.01	(0.58-1.76)	13	1.06	(0.61-1.85)	7	1.18	(0.56-2.49)	13	0.99	(0.57-1.72)	29	1.04	(0.72-1.51)	12	0.98	(0.55-1.74)
JEM2																						
Population																						
	Reference	683	1		644	1		629	1		411	1		698	1		698	1		683	1	
	School	66	0.83	(0.64-1.07)	66	0.84	(0.65-1.08)	48	0.81	(0.60-1.09)	38	0.76	(0.54-1.06)	67	0.83	(0.65-1.07)	67	0.83	(0.65-1.07)	32	0.76	(0.53-1.08)
Sex																						
	Female	288	1		278	1		264	1		180	1		296	1		296	1		276	1	
	Male	461	1.63	(1.40-1.90)	432	1.59	(1.36-1.86)	413	1.60	(1.36-1.88)	269	1.48	(1.21-1.80)	469	1.62	(1.39-1.88)	469	1.61	(1.39-1.88)	439	1.61	(1.38-1.89)
Asbestos exposure																						
	No	632	1		596	1		568	1		381	1		648	1		632	1		604	1	
	Yes	117	0.99	(0.80-1.22)	114	1.00	(0.81-1.23)	109	1.01	(0.81-1.25)	68	1.15	(0.88-1.51)	117	0.99	(0.81-1.22)	133	1.00	(0.83-1.22)	111	1.01	(0.82-1.24)
JEM2 >10 years																						
Population																						
	Reference	683	1		644	1		629	1		411	1		698	1		698	1		683	1	
	School	66	0.83	(0.64-1.07)	66	0.84	(0.65-1.08)	48	0.81	(0.60-1.08)	38	0.76	(0.54-1.05)	67	0.83	(0.65-1.07)	67	0.83	(0.65-1.07)	32	0.76	(0.53-1.08)
Sex																						
	Female	288	1		278	1		264	1		180	1		296	1		296	1		276	1	
	Male	461	1.62	(1.40-1.88)	432	1.58	(1.36-1.84)	413	1.60	(1.37-1.86)	269	1.49	(1.23-1.80)	469	1.61	(1.39-1.86)	469	1.61	(1.39-1.86)	439	1.60	(1.38-1.86)
Asbestos exposure																						
	No	734	1		695	1		663	1		439	1		750	1		734	1		700	1	
	Yes	15	1.18	(0.71-1.98)	15	1.22	(0.73-2.04)	14	1.20	(0.71-2.05)	10	2.31	(1.22-4.35)	15	1.18	(0.71-1.97)	31	1.13	(0.79-1.62)	15	1.31	(0.78-2.19)
JEM2 >15 years																						
Population																						
	Reference	683	1		644	1		629	1		411	1		698	1		698	1		683	1	
	School	66	0.83	(0.64-1.07)	66	0.84	(0.65-1.08)	48	0.81	(0.60-1.08)	38	0.76	(0.55-1.06)	67	0.83	(0.65-1.07)	67	0.83	(0.65-1.07)	32	0.76	(0.53-1.08)
Sex																						
	Female	288	1		278	1		264	1		180	1		296	1		296	1		276	1	
	Male	461	1.62	(1.40-1.88)	432	1.58	(1.36-1.84)	413	1.60	(1.37-1.86)	269	1.50	(1.24-1.81)	469	1.61	(1.39-1.86)	469	1.61	(1.39-1.87)	439	1.60	(1.38-1.87)
Asbestos exposure																						
	No	740	1		701	1		668	1		442	1		756	1		740	1		706	1	
	Yes	9	1.30	(0.67-2.51)	9	1.33	(0.69-2.58)	9	1.42	(0.73-2.75)	7	2.99	(1.41-6.34)	9	1.30	(0.67-2.51)	25	1.16	(0.78-1.72)	9	1.45	(0.75-2.81)

Online resource 3: Sensitivity analyses: Cox regression of leukaemia as first cancer, with other cancers and death as competing-risk, with different job exposure matrix models for occupational asbestos exposure adjustment.

		From Table 3 or 4			First			Second			Third			Fourth			Fifth			Sixth		
n		114,214			104,301			99,780			85,614			121,098			121,098			108,217		
					Born in Denmark (DK)			Schoolchildren born in			Born after January 1946			ATP missing into no			ATP missing into			Children from the two		
JEM1		Cases	HR	(95% CI)	Cases	HR	(95% CI)	Cases	HR	(95% CI)	Cases	HR	(95% CI)	Cases	HR	(95% CI)	Cases	HR	(95% CI)	Cases	HR	(95% CI)
Population																						
	Reference	303	1		289	1		287	1		173	1		314	1		314	1		303	1	
	School	38	1.07	(0.76-1.50)	37	1.04	(0.74-1.47)	27	1.00	(0.68-1.49)	22	1.02	(0.66-1.60)	41	1.12	(0.81-1.56)	41	1.11	(0.80-1.54)	25	1.33	(0.88-2.00)
Sex																						
	Female	140	1		133	1		130	1		86	1		148	1		148	1		136	1	
	Male	201	1.44	(1.15-1.80)	193	1.47	(1.17-1.85)	184	1.45	(1.15-1.83)	109	1.22	(0.90-1.63)	207	1.41	(1.14-1.76)	207	1.37	(1.10-1.71)	192	1.41	(1.12-1.77)
Asbestos exposure																						
	No	288	1		274	1		267	1		162	1		302	1		288	1		278	1	
	Yes	53	1.07	(0.79-1.46)	52	1.07	(0.78-1.45)	47	1.00	(0.73-1.38)	33	1.36	(0.92-2.02)	53	1.05	(0.78-1.43)	67	1.23	(0.93-1.62)	50	1.08	(0.79-1.48)
JEM1 >10 years																						
Population																						
	Reference	303	1		289	1		287	1		173	1		314	1		314	1		303	1	
	School	38	1.06	(0.76-1.49)	37	1.04	(0.74-1.46)	27	0.99	(0.66-1.47)	22	1.04	(0.66-1.62)	41	1.12	(0.81-1.55)	41	1.12	(0.81-1.55)	25	1.32	(0.88-1.99)
Sex																						
	Female	140	1		133	1		130	1		86	1		148	1		148	1		136	1	
	Male	201	1.42	(1.14-1.77)	193	1.44	(1.15-1.81)	184	1.41	(1.12-1.76)	109	1.24	(0.93-1.65)	207	1.39	(1.12-1.72)	207	1.38	(1.12-1.71)	192	1.39	(1.11-1.73)
Asbestos exposure																						
	No	326	1		311	1		299	1		186	1		340	1		326	1		313	1	
	Yes	15	1.78	(1.05-3.00)	15	1.79	(1.06-3.03)	15	1.89	(1.12-3.19)	9	2.51	(1.27-4.94)	15	1.75	(1.04-2.95)	29	2.00	(1.37-2.94)	15	1.93	(1.14-3.26)
JEM1 >15 years																						
Population																						
	Reference	303	1		289	1		287	1		173	1		314	1		314	1		303	1	
	School	38	1.07	(0.76-1.50)	37	1.04	(0.74-1.47)	27	1.00	(0.67-1.48)	22	1.05	(0.67-1.64)	41	1.13	(0.81-1.56)	41	1.13	(0.82-1.57)	25	1.33	(0.89-2.00)
Sex																						
	Female	140	1		133	1		130	1		86	1		148	1		148	1		136	1	
	Male	201	1.44	(1.16-1.79)	193	1.47	(1.17-1.83)	184	1.43	(1.14-1.79)	109	1.24	(0.93-1.65)	207	1.41	(1.14-1.74)	207	1.40	(1.13-1.73)	192	1.41	(1.13-1.76)
Asbestos exposure																						
	No	332	1		317	1		305	1		187	1		346	1		332	1		319	1	
	Yes	9	1.56	(0.80-3.04)	9	1.56	(0.80-3.04)	9	1.64	(0.84-3.19)	8	3.41	(1.67-6.98)	9	1.54	(0.79-3.00)	23	1.94	(1.27-2.96)	9	1.68	(0.86-3.27)
JEM2																						
Population																						
	Reference	303	1		289	1		287	1		173	1		314	1		314	1		303	1	
	School	38	1.06	(0.76-1.49)	37	1.04	(0.74-1.46)	27	1.00	(0.67-1.48)	22	1.04	(0.66-1.61)	41	1.12	(0.81-1.55)	41	1.11	(0.80-1.54)	25	1.32	(0.88-1.99)
Sex																						
	Female	140	1		133	1		130	1		86	1		148	1		148	1		136	1	
	Male	201	1.41	(1.12-1.77)	193	1.43	(1.13-1.81)	184	1.43	(1.13-1.81)	109	1.24	(0.92-1.67)	207	1.38	(1.11-1.72)	207	1.34	(1.07-1.67)	192	1.39	(1.10-1.75)
Asbestos exposure																						
	No	281	1		267	1		262	1		165	1		295	1		281	1		272	1	
	Yes	60	1.18	(0.88-1.59)	59	1.18	(0.87-1.59)	52	1.07	(0.78-1.46)	30	1.24	(0.82-1.87)	60	1.16	(0.87-1.55)	74	1.33	(1.01-1.74)	56	1.16	(0.86-1.58)
JEM2 >10 years																						
Population																						
	Reference	303	1		289	1		287	1		173	1		314	1		314	1		303	1	
	School	38	1.05	(0.75-1.48)	37	1.03	(0.73-1.45)	27	0.98	(0.66-1.46)	22	1.03	(0.66-1.60)	41	1.11	(0.80-1.54)	41	1.12	(0.81-1.55)	25	1.31	(0.87-1.97)
Sex																						
	Female	140	1		133	1		130	1		86	1		148	1		148	1		136	1	
	Male	201	1.42	(1.14-1.77)	193	1.44	(1.15-1.80)	184	1.41	(1.13-1.77)	109	1.25	(0.94-1.66)	207	1.39	(1.12-1.72)	207	1.39	(1.12-1.72)	192	1.39	(1.11-1.73)
Asbestos exposure																						
	No	329	1		314	1		303	1		189	1		343	1		329	1		316	1	
	Yes	12	2.14	(1.19-3.84)	12	2.16	(1.20-3.88)	11	2.09	(1.14-3.85)	6	3.35	(1.47-7.62)	12	2.11	(1.18-3.78)	26	2.23	(1.49-3.33)	12	2.35	(1.31-4.22)
JEM2 >15 years																						
Population																						
	Reference	303	1		289	1		287	1		173	1		314	1		314	1		303	1	
	School	38	1.06	(0.76-1.48)	37	1.03	(0.73-1.45)	27	0.99	(0.67-1.47)	22	1.04	(0.67-1.62)	41	1.11	(0.80-1.54)	41	1.13	(0.81-1.56)	25	1.32	(0.88-1.99)
Sex																						
	Female	140	1		133	1		130	1		86	1		148	1		148	1		136	1	
	Male	201	1.43	(1.15-1.78)	193	1.45	(1.16-1.82)	184	1.42	(1.14-1.79)	109	1.25	(0.94-1.67)	207	1.40	(1.13-1.73)	207	1.41	(1.14-1.74)	192	1.40	(1.12-1.75)
Asbestos exposure																						
	No	333	1		318	1		307	1		190	1		347	1		333	1		320	1	
	Yes	8	2.59	(1.28-5.26)	8	2.60	(1.28-5.27)	7	2.41	(1.13-5.13)	5	5.25	(2.14-12.86)	8	2.56	(1.26-5.19)	22	2.39	(1.55-3.69)	8	2.89	(1.43-5.87)
